# Circ_0064288 acts as an oncogene of hepatocellular carcinoma cells by inhibiting miR-335-5p expression and promoting ROCK1 expression

**DOI:** 10.1186/s12885-022-09323-8

**Published:** 2022-03-14

**Authors:** Yingying Nie, Xuedan Zhu, Nan Bu, Yang Jiang, Yue Su, Keming Pan, Shanshan Li

**Affiliations:** 1 Department of Gastroenterology, Jiamusi Central Hospital, Jiamusi, 154002 Heilongjiang China; 2 Department of Gastroenterology, the First Affiliated Hospital of Harbin Medical University, Harbin, 150000 Heilongjiang China; 3 Department of Gastroenterology, Jiamusi Hospital of Traditional Chinese Medicine, No.326 Jiefang Road, Jiamusi, 154002 Heilongjiang China

**Keywords:** HCC, circ_0064288, miR-335-5p, ROCK1

## Abstract

**Background:**

Reportedly, circular RNA (circRNA) is a key modulator in the development of human malignancies. This work is aimed to probe the expression pattern, biological effects and mechanism of circ_0064288 on hepatocellular carcinoma (HCC) progression.

**Methods:**

The differentially expressed circRNA was screened by analyzing the expression profiles of circRNAs in HCC tissues and normal tissues. Quantitative real-time polymerase chain reaction (qRT-PCR) was performed to examine the expression of circ_0064288, miR-335-5p and Rho associated coiled-coil containing protein kinase 1 (ROCK1) mRNA in HCC specimens. After circ_0064288 was overexpressed or knocked down in HCC cells, cell growth was detected by the CCK-8 experiment, and cell migration was evaluated using Transwell experiment and scratch healing experiment. The targeting relationship between miR-335-5p and circ_0064288 and ROCK1 mRNA was predicted and verified using bioinformatic analysis and dual-luciferase reporter gene experiments, respectively. Western blot was executed to examine ROCK1 protein expression in HCC cells.

**Results:**

Circ_0064288 and ROCK1 expression was up-modulated in HCC, while miR-335-5p was down-modulated. High circ_0064288 expression was associated with shorter survival time of HCC patients. It was also revealed that circ_0064288 overexpression remarkably enhanced HCC cell growth and migration, while knockdown of circ_0064288 induced opposite effects. Additionally, circ_0064288 could competitively bind with miR-335-5p thereby up-modulate ROCK1 expression. MiR-335-5p overexpression partly counteracted the effect of circ_0064288 overexpression on HCC cells.

**Conclusion:**

Circ_0064288 facilitates HCC cell growth and migration by modulating the miR-335-5p/ROCK1 axis.

**Supplementary Information:**

The online version contains supplementary material available at 10.1186/s12885-022-09323-8.

## Introduction

Hepatocellular carcinoma (HCC) is the most common histological subtype of liver cancer, taking up around 90% of primary liver cancer cases [1]. Statistically, HCC is the third major cause of cancer-related death worldwide [2]. Although hepatectomy, liver transplantation, chemotherapy and radiotherapy can extend the survival of HCC patients to some extent, the high aggressiveness and recurrence rates of HCC still result in adverse prognosis for most patients [3]. Hence, it is imperative to clarify the molecular mechanisms of HCC.

Circular RNAs (circRNAs) are a class of non-coding RNA transcripts that possess a covalent closed-loop structure, formed by reverse splicing of precursor mRNA (pre-mRNA), and do not possess the 5′ end cap structure and 3′ end polyadenylate tail, and the closed-loop structure makes them resistant to cleavage by ribonucleic acid exonuclease, so they are much more stable than liner RNA [4]. The specific expression characteristics and biological functions of circRNAs in tumors confer their potential as tumor biomarkers and therapeutic targets [5, 6]. For instance, circ_0000517 expression is higher in HCC tissues than in paracancerous tissues, and high circ_0000517 expression is linked to unfavorable prognosis in HCC patients [5]. However, the expression characteristics and biological function of most circRNAs in HCC are still undefined.

MicroRNAs (miRNAs) are highly conserved and single-stranded small non-coding RNAs, and these small molecules influence almost all the aspects of biological processes [7]. A lot of miRNAs are reported to participate in the carcinogenesis and progression of HCC [8]. Recent research unveils that circRNAs can work as molecular sponges of miRNAs, and modulate mRNA translation by decoying miRNAs [9]. For instance, circ_0001955 enhances HCC tumorigenesis by adsorbing miR-516a-5p to promote the expression of TRAF6 and MAPK11 [10].

Circ_0064288, also known as circRNA_103285, was generated from the transcript of autophagy related 7 (ATG7), which is reported to be up-regulated in HCC samples [11]. In this work, two Gene Expression Omnibus (GEO) datasets (GSE94508 and GSE97332) were analyzed to look for the differentially expressed circRNAs in HCC, and it was revealed that circ_0064288 was a potential up-regulated circRNA in HCC. We hypothesized that circ_0064288 was an oncogenic circRNA in HCC. Interestingly, bioinformatics analysis suggested that miR-335-5p was a potential downstream target of circ_0064288, and Rho associated coiled-coil containing protein kinase 1 (ROCK1) was a potential downstream target of miR-335-5p. This study was performed to clarify the expression pattern, biological function and the downstream competitive endogenous RNA (ceRNA) mechanism of circ_0064288 in HCC. Herein, we report that circ_0064288, which is highly expressed in HCC, acts as an oncogene by inhibiting miR-335-5p expression and promoting ROCK1 expression.

## Materials & methods

### Tissue samples

HCC tissues and paracancerous tissues were collected from 36 patients who received hepatectomy in Jiamusi Central Hospital from May 2012 to April 2014. All subjects did not received radiotherapy or chemotherapy before undergoing tumor resection. The research was endorsed by the Ethics Committee of Jiamusi Central Hospital and written informed consent was acquired from each subject.

### Acquisition of GEO data

GEO database is a public database that provides access to gene expression datasets. CircRNA microarray datasets of HCC were searched using the following keywords “circRNA” and “liver cancer”, and GSE94508 and GSE97332 were obtained. The corresponding raw data were then downloaded and analyzed with GEO2R online analysis tool. The circRNAs with *P* < 0.05 and │log2(Fold Change)│ > 1 in each data set were regarded the differentially expressed circRNAs.

### Cell culture and transfection

Human HCC cell lines (Huh7, Hep3B, HCCLM3, and MHCC97-L) and human normal liver epithelial cell line (THLE-3) were procured from American Type Culture Collection (ATCC, Manassas, VA, USA) and Shanghai Cell Bank of Chinese Academy of Sciences (Shanghai, China). All cells were cultured in RPMI-1640 medium (Gibco, Carlsbad, CA, USA) containing 10% fetal bovine serum (FBS) (Gibco, Carlsbad, CA, USA) and 100 U/mL penicillin, and 0.1 mg/mL streptomycin (Invitrogen, Carlsbad, CA, USA) at 37 °C with 5% CO_2_. Circ_0064288 overexpression plasmid pcDNA3.1-circ_0064288 (circ_0064288), empty vector (NC), circ_0064288 siRNA (si-circ_0064288#1 and si-circ_ circ_0064288#2), scramble siRNA (si-NC), miR-335-5p mimics/miR-335-5p inhibitors and negative controls (mimics NC/inhibitors NC) were procured from Invitrogen (Carlsbad, CA, USA). Transient transfection was performed with Lipofectamine® 2000 (Invitrogen, Carlsbad, CA, USA) according to the manufacturer’s direction .

### Quantitative real-time polymerase chain reaction (qRT-PCR)

Homogenized HCC tissue and cells were collected, and total RNA was extracted using TRIzol Reagent (Yeasen Biotech, Shanghai, China). cDNA synthesis was implemented using the TaqMan MiRNA reverse transcription kit (Applied Biosystems, Foster City, CA, USA) for miR-335-5p and employing a PrimeScript RT Master Mix Kit (TaKaRa, Dalian, China) for ROCK1 and circ_0064288, respectively. Then with cDNA as the template, qRT-PCR was implemented to determine the relative expressions of ROCK1 and circ_0064288 with the SYBR® Premix Ex Taq™ II kit (TaKaRa, Dalian, China). Meanwhile, a stem-loop primer SYBR Green qRT-PCR kit (Synbio Tech, Suzhou, China) was used for qRT-PCR to evaluate miR-335-5p expression. With U6 and GAPDH as internal references, the relative expressions of circ_0064288, miR-335-5p and ROCK1 were calculated by 2^−ΔΔCt^ method. The primer sequences are listed in Table 1. The cytoplasm and nuclear fractions of the HCC cells were separated using a PARIS™ Kit (Invitrogen, Carlsbad, CA, USA) following the manufacturer’s instructions, and next, the RNA in cytoplasm and nuclei was respectively extracted. Then, the expression of circ_0064288 in the cytoplasm and nucleus was measured by qRT-PCR. GAPDH and U6 were used as cytoplasm and nuclear controls, respectively.

**Table 1:** Primer sequences

Name	Primer sequences
circ_0064288	Forward: 5′-TGGAACAAGCAGCAAATGAG-3′
	Reverse: 5′-AATAGCTGGGCAGCAACG-3′
miR-335-5p	Forward: 5′-UGUUUUGAGCGGGGGUCAAGAGC-3’
	Reverse: 5′-CUCUCAUUUGCUAUAUUCA-3′
ROCK1	Forward: 5′-AACATGCTGCTGGATAAATCTGG-3′
	Reverse: 5′-TGTATCACATCGTACCATGCCT-3′
U6	Forward: 5′-CTCGCTTCGGCAGCACA-3’
	Reverse: 5′-AACGCTTCACGAATTTGCGT-3’
GAPDH	Forward: 5′-GGGAAACTGTGGCGTGAT-3’
	Reverse: 5′-GAGTGGGTGTCGCTGTTGA-3’

### CCK-8 experiment

Transfected HCC cells were planted in 96-well plates (2 × 10^3^ cells/well) containing 100 μL of medium/well. 10 μL of CCK-8 solution (Beyotime, Shanghai, China) was supplemented to each well at the indicated time points (0 h, 24 h, 48 h, 72 h, 96 h). Subsequently, the cells were incubated at 37 °C for 2 h and the absorbance at 450 nm was measured with a microplate reader.

### Transwell experiment

Transwell chambers (Corning Life Sciences, Corning, NY, USA) were utilized to detect the migration of HCC cells. The cells of each group in the logarithmic growth period were taken, trypsinized with 0.25% trypsin and re-suspended in serum-free medium, and the cell concentration was modulated to 1 × 10^5^ cell/ml. In the top compartment, 200 μL of cell suspension was supplemented, and 500 μL of medium containing 10% FBS was added into the bottom compartment, and subsequently, the cells were cultured for 24 h. Next, methanol-fixed migrated cells were stained with 0.1% crystal violet. Five fields of view were randomly selected under a microscope, and the cells were counted, and the average value was calculated.

### Wound healing experiment

HCC cells were planted in 6-well plates (1 × 10^4^ cells/well) and cultured, and when the cells were spread all over the plates, the tips of 200 μL sterile pipette tubes were used to make scratches in the middle. The cells were then gently rinsed 3 times with serum-free medium, and then cultured with serum-free medium. Photographs were taken at 0 h and 24 h after scratch formation and the width of the scratch was recorded.

### Western blot

Transfected HCC cells were lysed using RIPA lysis buffer (Beyotime, Shanghai, China), the supernatant of the lysate was harvested to collect the total protein. A BCA protein assay kit (Beyotime, Haimen, China) was applied to determine protein concentration. Proteins were separated by sodium dodecyl sulfate polyacrylamide gel electrophoresis (SDS-PAGE) and transferred to polyvinylidene fluoride (PVDF) membrane. 5% skim milk was adopted to block the membrane for 1 h at room temperature. Subsequently, anti-ROCK1 antibody (1:1000, ab134181, Cambridge, UK) and anti-GAPDH antibody (1:2000, ab8245, Cambridge, UK) were applied to incubate the PVDF membrane at 4 °C overnight. The next day, the membranes were incubated with horseradish peroxidase-conjugated secondary antibodies (1:5000, Beyotime, Shanghai, China) for 50 min at room temperature. The protein bands were developed using an Amersham Imager 600 (GEHealthcare, Chicago, IL, USA) with an electrochemiluminescence kit (Biosharp, Hefei, China).

### Luciferase reporter experiment

StarBase database was utilized to predict the binding sites between miR-335-5p and circ_0064288 / ROCK1 3’UTR, respectively, and the binding fragments of circ_0064288 and ROCK1 3’UTR were amplified by PCR. The amplification products were inserted into the PGL3-promoter plasmid vector (Promega, Madison, WI, USA) to construct the circ_0064288 and ROCK1 wild-type (WT) plasmids; the binding fragment was mutated using gene mutation technology to construct the circ_0064288 and ROCK1 mutant type (MUT) plasmid. The recombinant plasmids were co-transfected with miR-335-5p mimic (or mimic NC) respectively in HEK-293 T cells. After 48 h, the cells were collected. Fluorescence values were measured using a dual-luciferase reporter gene assay system (Promega, Madison, WI, USA).

### Statistical analysis

All of the experiments were performed at least in triplicate, and at least repeated for 3 times. All data were expressed as “mean ± SD”, and GraphPad Prism 8 (GraphPad Software, Inc., La Jolla, CA, US) was employed for graphing and statistical analysis. Kolmogorov-Smirnov test was used to examine the normality and equal variance of the data. Student’s t-test was utilized to make the comparison between the two groups. One-way ANOVA and Dunnett’s post-hoc multiple comparisons were employed for comparisons among three or more groups. For data that were skewed distributed, comparisons between two groups were performed by Wilcoxon signed-rank test. Chi-square test was employed to elucidate the correlation between LINC00518 expression and pathological parameters. Kaplan-Meier survival curve and log-rank test were adopted to compare patient’s prognosis in different groups. Gene expression correlations were analyzed by Pearson correlation coefficient. *P* < 0.05signified statistical significance.

## Results

### Circ_0064288 is overexpressed in HCC

**Fig. 1:**
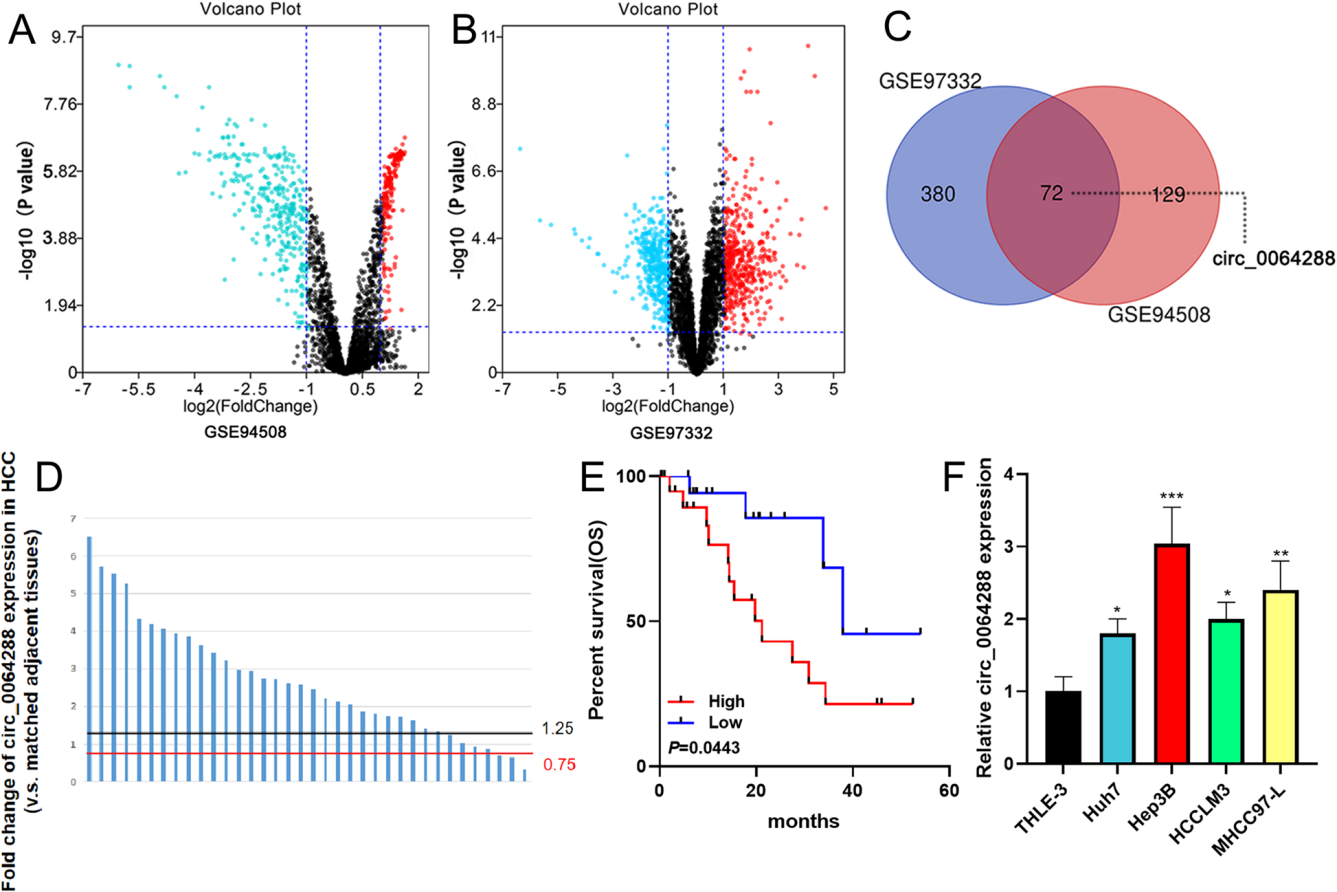
Circ_0064288 expression is up-modulated in HCC. **A**-**B**. Volcano plots were utilized to show the differentially expressed circRNAs in HCC, with the data of GSE94508 and GSE97332. **C**. Venn diagram was employed to screen the circRNAs that were remarkably up-modulated in both datasets. **D**. Circ_0064288 expression in HCC tissues and paracancerous tissues was examined by qRT-PCR, and then the fold change of circ_0064288 expression in each HCC sample was shown. **E**. Kaplan-Meier curve was applied to analyze the relationship between circ_0064288 expression and the overall survival of HCC patients. **F**. Circ_0064288 expression in normal hepatic epithelial cell line (THLE-3) and HCC cell lines (Huh7, Hep3B, HCCLM3, and MHCC97-L) was measured by qRT-PCR. * *P* < 0.05, ** *P* < 0.01, ***P < 0.001

GSE94508 and GSE97332 were obtained from the GEO database, and circRNAs’ expression levels in HCC tissues and adjacent tissues were analyzed online using GEO2R tool. The volcano plot showed the expression patterns of the circRNAs in the datasets (Fig. 1A-B). 72 circRNAs were up-modulated in both datasets, and circ_0064288 was among them (log_2_ fold change = 1.11 and 1.14, respectively) (Fig. 1C, Table 2, Supplementary Table 1, Supplementary Table 2). qRT-PCR was executed to examine circ_0064288 expression in adjacent liver tissues and HCC tissue specimens from 36 HCC patients, and the data showed that in most HCC patients (29 of the 36 patients), the expression of circ_0064288 was significantly up-modulated in the cancerous tissues (fold change > 1.25, v.s. matched adjacent liver tissues) (Fig. 1D). Additionally, the 36 patients mentioned above were divided into circ_0064288 high expression group and low expression group (according to the fold change of expression level v.s. adjacent liver tissues), and the relationship between the clinical characteristics of the patients and the expression level of circ_0064288 was analyzed, and it was revealed that higher expression of circ_0064288 was associated with larger tumor size of the patients (*P* = 0.042, Table 3). Furthermore, a Kaplan-Meier survival curve was plotted, and log-rank test showed that high circ_0064288 expression was remarkably with shorter overall survival time of the HCC patients (*P* = 0.0443, Fig. 1E). Besides, circ_0064288 expression was markedly augmented in HCC cell lines [Huh7 (*P*<0.001), Hep3B (*P*<0.001), HCCLM3 (*P*<0.001), and MHCC97-L (*P*<0.001)] relative to normal hepatic epithelial cell line (THLE-3) (Fig. 1F).

**Table 2:** The up-regulated circRNAs in HCC in both GSE97332 and GSE94508

Up-regulated circRNAs	
circRNA_101441, circRNA_103316, circRNA_102148, circRNA_101798, circRNA_101313, circRNA_400087, circRNA_103348, circRNA_103563, circRNA_100090, circRNA_103809, circRNA_102559, circRNA_101051, circRNA_104268, circRNA_103038, circRNA_102034, circRNA_104575, circRNA_104760, circRNA_101975, circRNA_101144, circRNA_101707, circRNA_102954, circRNA_104993, circRNA_000872, circRNA_104017, circRNA_100571, circRNA_101094, circRNA_001416, circRNA_104003, circRNA_103546, circRNA_400071, circRNA_103910, circRNA_000792, circRNA_400091 circRNA_101287, circRNA_104499, circRNA_000996, **circRNA_103285**, circRNA_000764, circRNA_101555, circRNA_101571, circRNA_102205, circRNA_105024, circRNA_103948, circRNA_102451, circRNA_102430, circRNA_101201, circRNA_101408, circRNA_101141, circRNA_102644, circRNA_104640, circRNA_103380, circRNA_100192, circRNA_000481, circRNA_104336, circRNA_102482, circRNA_104771, circRNA_101777, circRNA_100445, circRNA_000956, circRNA_102587, circRNA_100053, circRNA_103213, circRNA_104475, circRNA_001059, circRNA_104833, circRNA_001067, circRNA_001506, circRNA_400048, circRNA_103188, circRNA_105027, circRNA_100367, circRNA_101748.

**Table 3:** Correlation between circ_0064288 expression and clinicopathological features of the 36 HCC patients

Characteristics	Number	circ_00064288 expression		***P*** value
		Low	High	
Age (years)				
≤ 50	9	3	6	0.441
> 50	27	15	12	
Gender				
Male	22	11	11	1.000
Female	14	7	7	
TMN stage				
I/II	13	9	4	0.165
III/IV	23	9	14	
Tumor size (cm)				
< 5	15	11	4	0.042*
≥ 5	21	7	14	
Vascular invasion				
Yes	9	2	7	0.124
No	27	16	11	
AFP (μg/L)				
≤ 400	11	6	5	1.000
> 400	25	12	13	
Aetiology				
HBV infection	26	13	13	0.435
Alcoholic liver disease	4	3	1	
Fatty liver disease & others	6	2	4	

*P* value was calculated by Fisher’s exact test. * denotes *p* values less than 0.05

### Circ_0064288 contributes to the multiplication and migration of HCC cells

**Fig. 2:**
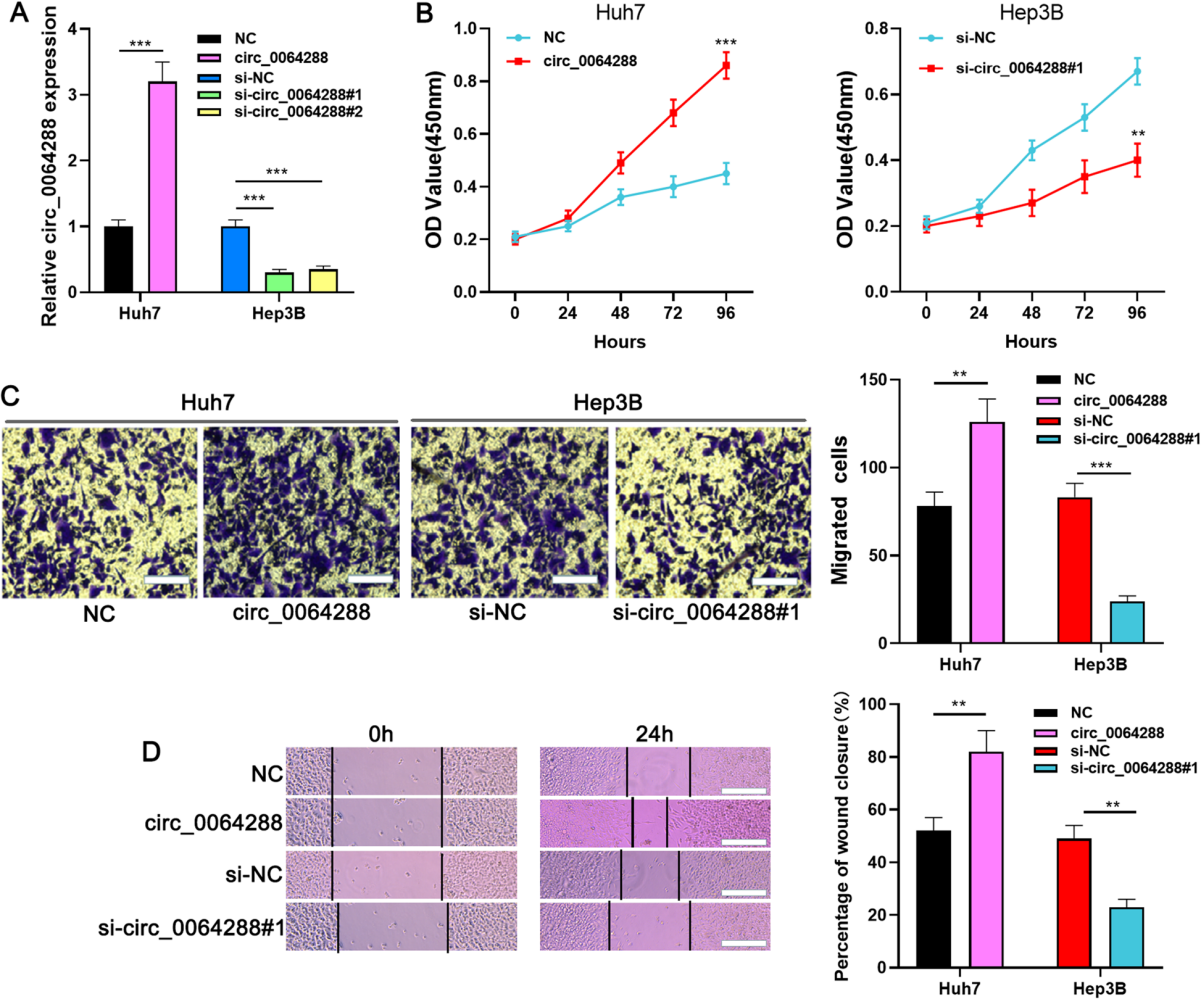
Circ_0064288 accelerates the growth and migration of HCC cells. **A**. Circ_0064288 overexpression plasmid (circ_0064288) and circ_0064288 siRNAs (si-circ_0064288#1 or si-circ_0064288#2) and the corresponding controls (NC or si-NC) were transfected into Huh7 and Hep3B cells, respectively, and the transfection efficiency was detected by qRT-PCR. **B**. CCK-8 experiment was adopted to detect the growth of HCC cells after transfection. **C**-**D**. Transwell assay (scale bar, 25 μm) and scratch healing assay (scale bar, 100 μm) were utilized to examine the migration of HCC cells after transfection. ** *P* < 0.01, *** *P* < 0.001

Among the four cell lines, the expression of circ_0064288 was the lowest in Huh7 cells (fold change was about 1.8, v.s. THLE-3 cells), but the highest in Hep3B cells (fold change was about 3, v.s. THLE-3 cells), so circ_0064288 was overexpressed in Huh7 cells, and it was knocked down in Hep3B cell (Fig. 1E). circ_0064288 overexpression plasmid and circ_0064288 siRNA (si-circ_0064288#1 and si-circ_0064288#2) and the corresponding controls (NC or si-NC) were transfected into Huh7 and Hep3B cells, respectively, to construct circ_0064288 overexpression and circ_0064288 knockdown cell models (Fig. 2A). si-circ_0064288#1 knockdown efficiency was higher (knockdown efficiency of about 80% in si-circ_0064288#1 group, knockdown efficiency of about about 70% in si-circ_0064288#1 group), so si-circ_0064288#1 was selected for subsequent experiments (Fig. 2A). The data of CCK-8 experiment revealed that the cell growth in circ_0064288 overexpression group was notably promoted than that of the control group (*P*<0.001) (Fig. 2B). Moreover, in Transwell assay, after circ_0064288 was overexpressed, the number of Huh7 cells which passed the filter was increased (fold change ≈ 1.6, *P*<0.01, v.s. the control group); in wound-healing assay, the speed of scratch healing in circ_0064288 overexpression group is also higher (fold change ≈ 1.6, *P*<0.01, v.s. the control group) (Fig. 2C-D). Furthermore, knockdown of circ_0064288 reduced the growth of Hep3B cells (*P*<0.001, v.s. the control group), the number of Hep3B cells which passed the filter (fold change ≈ 0.25, *P*<0.001, v.s. the control group), and the speed of scratch healing of Hep3B cells (fold change ≈ 0.5, *P*<0.01, v.s. the control group) (Fig. 2B-D). These experiments suggested that circ_0064288 worked as an oncogenic factor in HCC.

### Circ_0064288 functions as ceRNA and sponges miR-335-5p

**Fig. 3:**
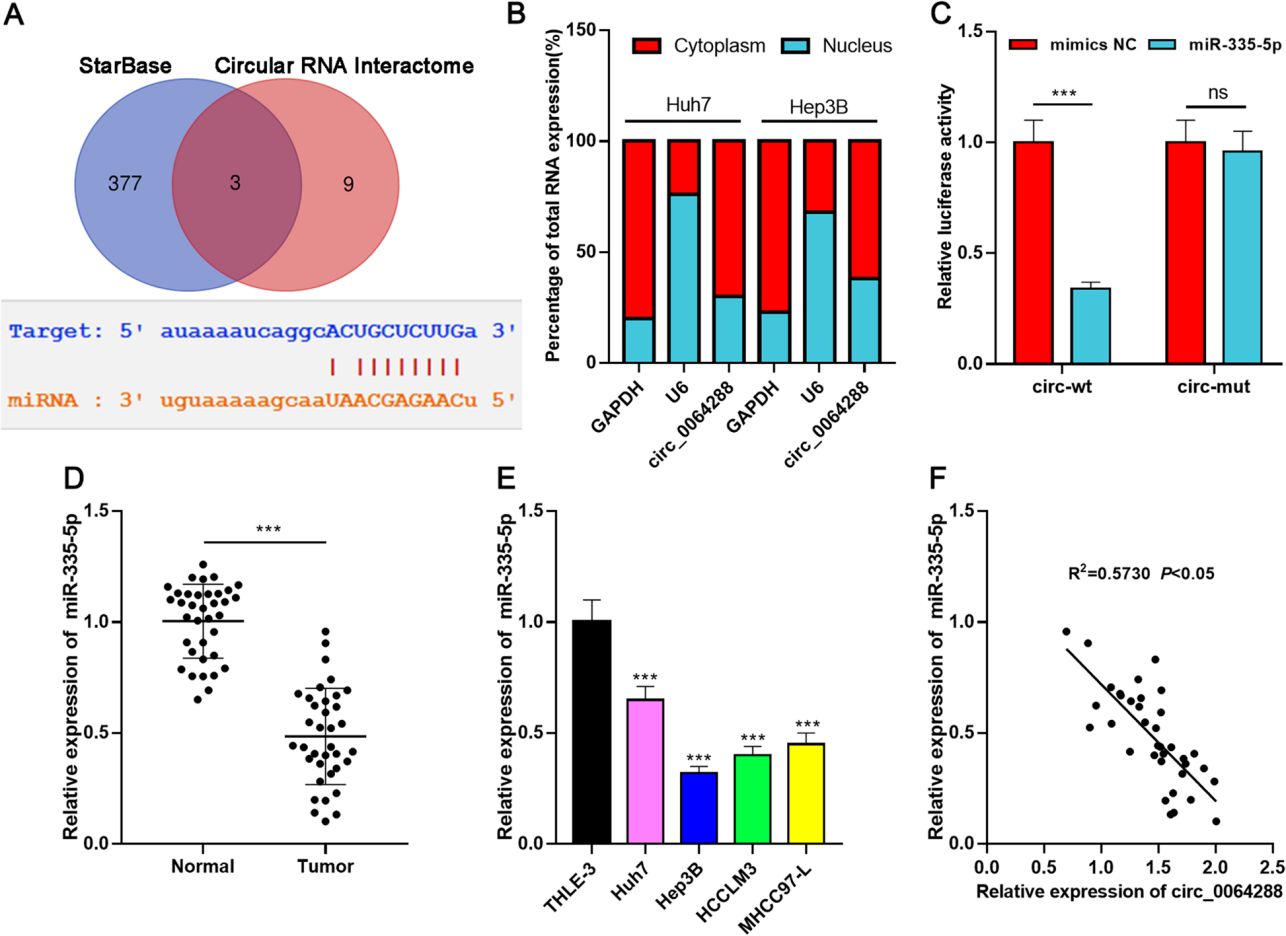
Circ_0064288 functions as ceRNA and sponges miR-335-5p. **A**. Bioinformatics databases (StarBase and Circular RNA Interactome) were used to predict the binding site of circ_0064288 to miR-335-5p. **B**. qRT-PCR was employed to detect circ_0064288 expression in the cytoplasm and nucleus of Huh7 and Hep3B cells. **C**. Dual-luciferase reporter gene experiment showed that the predicted binding sequence between circ_0064288 and miR-335-5p was functional. **D**. qRT-PCR was employed to detect miR-335-5p expression in HCC tissues and paracancerous tissues. **E**. qRT-PCR was used to detect miR-335-5p expression in normal hepatic epithelial cell line (THLE-3) and HCC cell lines (Huh7, Hep3B, HCCLM3, and MHCC97-L). **F**. The correlation between circ_0064288 expression and miR-335-5p expression in HCC tissues was evaluated by Pearson’s correlation coefficient. ***P < 0.001, ns, means no statistical significance. ***P < 0.001, ns, means no statistical significance

Increasing research has reported that circRNAs act as ceRNA to regulate miRNAs in cancer biology [9, 12]. Candidate miRNA targets of circ_0064288 were analyzed online using two bioinformatics tools (StarBase and Circular RNA Interactome), and three common miRNAs (miR-335-5p, miR-498, miR-574-5p) were predicted by both of two bioinformatics tools (Fig. 3A). qRT-PCR revealed that circ_0064288 was mainly distributed in the cytoplasm, suggesting it could probably function as a ceRNA (Fig. 3B). Dual-luciferase reporter gene experiment demonstrated that miR-335-5p repressed the luciferase activity of circ_0064288-WT reporter (inhibition efficiency is about 70%, *P*<0.001), but exerted no remarkable effect on luciferase activity of circ_0064288-MUT reporter (*P >* 0.05) (Fig. 3C). Further, miR-335-5p expression in HCC was detected by qRT-PCR, and the data unveiled that miR-335-5p expression was remarkably lower in HCC tissues than in paracancerous tissues (*P*<0.001) (Fig. 3D). MiR-335-5p expression was remarkably weakened in HCC cell lines [Huh7 (*P*<0.001), Hep3B (*P*<0.001), HCCLM3 (*P*<0.001), and MHCC97-L (*P*<0.001)] relative to normal hepatic epithelial cell line (Fig. 3E). In addition, miR-335-5p expression was negatively correlated with circ_0064288 expression in HCC tissues (R^2^ = 0.5730, *P*<0.001) (Fig. 3F). Collectively, there was a binding relationship between miR-335-5p and circ_0064288, and there could probably be a regulatory relationship between them in HCC cells.

### MiR-335-5p counteracts the promoting effects of circ_0064288 on HCC progression

**Fig. 4:**
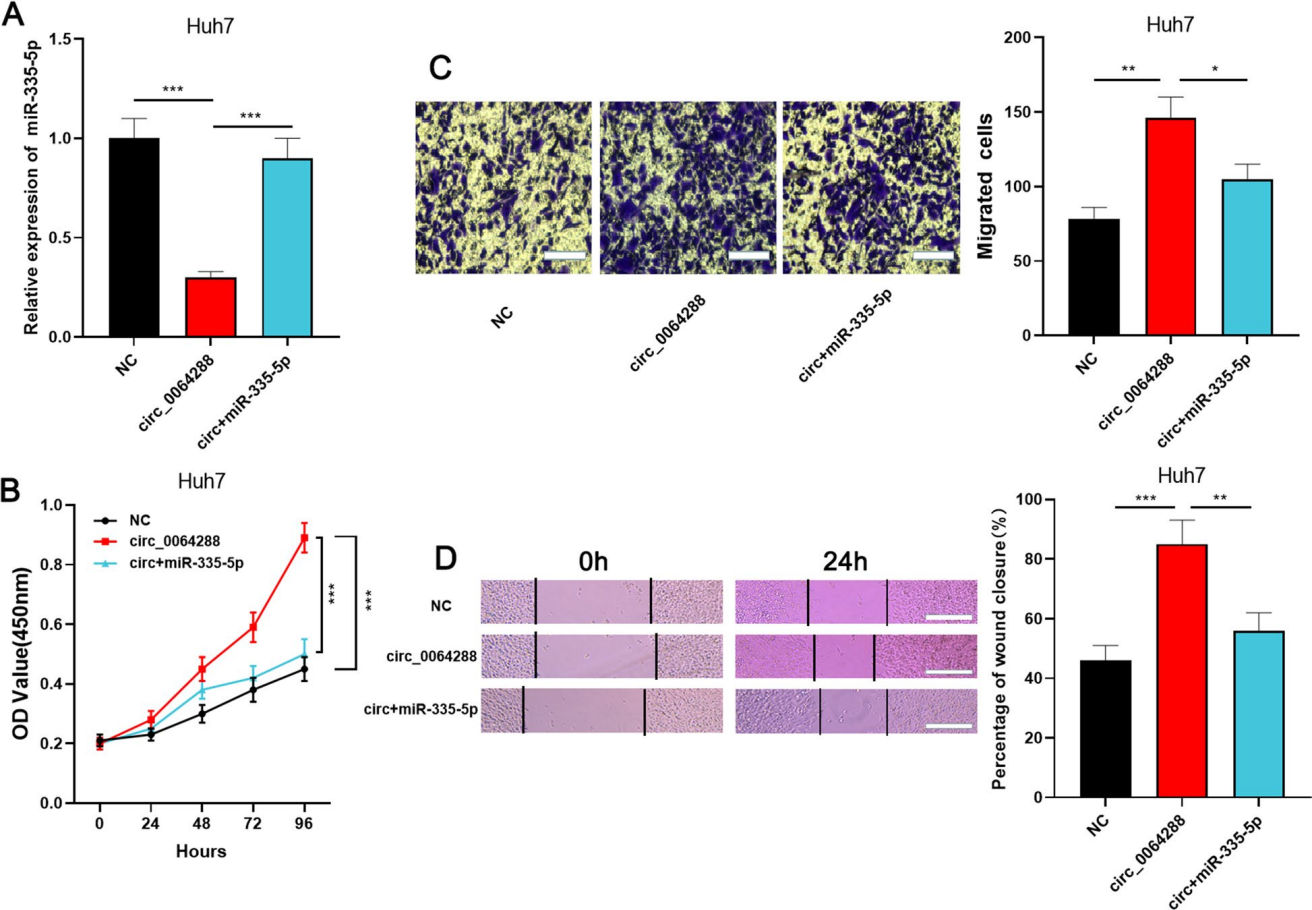
Overexpression of miR-335-5p counteracted the biological effects of circ_0064288 on HCC cells. **A**. Huh7 cells overexpressing circ_0064288 were transfected with miR-335-5p mimics, and the expression level of miR-335-5p in HCC cells was detected by qRT-PCR. **B**. CCK-8 assay was used to detect the proliferation of HCC cells after the transfection. **C**-**D**. Transwell assay (scale bar, 25 μm) and wound healing assay (scale bar, 100 μm) were used to detect the migration of HCC cells after the transfection. *P < 0.05, **P < 0.01, and ***P < 0.001

To probe the role of circ_0064288 and miR-335-5p in modulating the growth and migration of HCC cells, Huh7 cells overexpressing circ_0064288 were co-transfected with miR-335-5p mimics (Fig. 4A). The data of the rescue assay showed that the effect of circ_0064288 overexpression in facilitating the proliferation (*P*<0.001) and migration (*P*<0.05 in Transwell assay, *P*<0.01 in wound healing assay) of Huh7 cells was counteracted by miR-335-5p (Fig. 4B-D). The above results indicated that the effect of circ_0064288 in facilitating the malignant phenotypes of Huh7 cells was probably dependent on miR-335-5p.

### Circ_0064288 augmented ROCK1 expression in HCC cells by targeting miR-335-5p

**Fig. 5:**
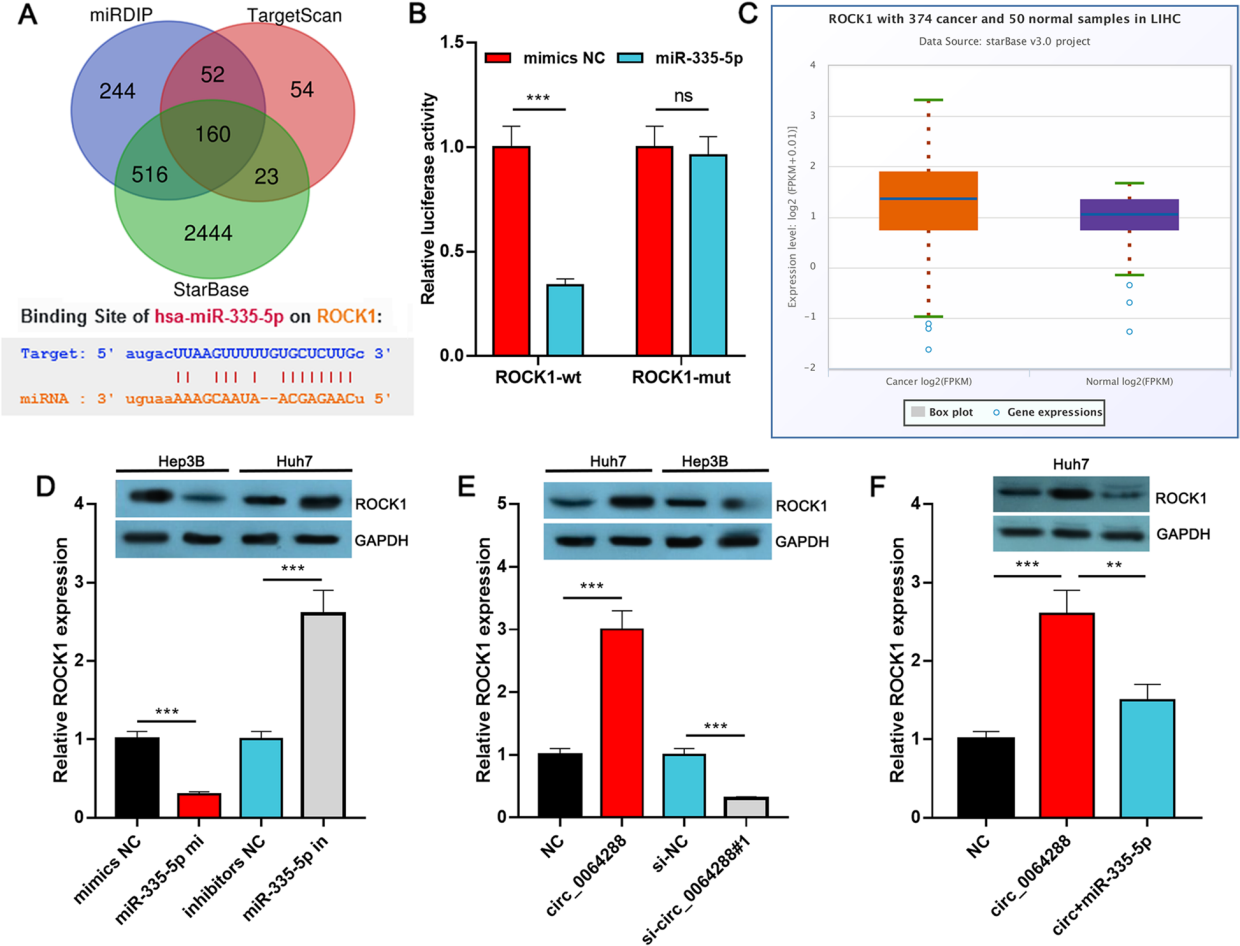
Circ_0064288 increases ROCK1 expression by targeting miR-335-5p. **A**. Three bioinformatics algorithms (miRDIP, StarBase and Targetscan) were used to predict the binding sites of miR-335-5p within the 3’UTR of ROCK1. **B**. Dual-luciferase reporter experiment was executed to confirm the predicted binding sequence between miR-335-5p and ROCK1 3’UTR. **C**. StarBase database was utilized to analyze ROCK1 expression in HCC tissues and paracancerous tissues. **D**-**F**. qRT-PCR and Western blot experiments were applied to detect ROCK1 expression in HCC cells after the expression of circ_0064288 and miR-335-5p were selectively regulated. **P < 0.01, ***P < 0.001, ns, means no statistical significance

With three bioinformatics algorithms including miRDIP, StarBase and Targetscan, we found that 160 potential target genes were predicted, including ROCK1 (Fig. 5A, Supplementary Table 3). Next, Gene Set Enrichment Analysis (GSEA) was performed, and results demonstrated that high ROCK expression is positively associated with the multiple signal pathways / biological processes, including DNA replication, cell cycle, Notch signal pathway and mTOR signal pathway (*P* < 0.001, and FDR < 0.25) (Supplementary Fig. 1A-D). Next, the targeting relationship between ROCK1 and miR-335-5p was confirmed by dual-luciferase reporter gene experiment: miR-335-5p repressed the luciferase activity of ROCK1-WT reporter (inhibition efficiency is about 70%, *P*<0.001), but exerted no remarkable effect on luciferase activity of ROCK1-MUT reporter (*P >* 0.05) (Fig. 5B). StarBase database showed that ROCK1 expression was remarkably higher in HCC tissues than in paracancerous tissues (fold change = 1.39, *P* = 0.0039) (Fig. 5C). qRT-PCR and Western blot experiments showed that transfection of miR-335-5p mimics remarkably repressed ROCK1 expression in Hep3B cells at protein level and mRNA level (fold change < 0.5, *P*<0.001); conversely, transfection of miR-335-5p inhibitors in Huh7 cells markedly augmented ROCK1 expression at protein level and mRNA level (fold change > 2.5, *P*<0.001) (Fig. 5D). Circ_0064288 overexpression induced an augmentation in ROCK1 expression in Huh7 cells at protein level and mRNA level (fold change ≈ 3, *P*<0.001), while knockdown of circ_0064288 restrained ROCK1 expression in Hep3B cells at protein level and mRNA level (fold change < 0.5, *P*<0.001) (Fig. 5E). Besides, circ_0064288 overexpression induced an increase in ROCK1 expression at protein level and mRNA level (fold change > 2.5, *P*<0.001), and that could be counteracted by the co-transfection of miR-335-5p mimics at protein level and mRNA level (fold change ≈ 0.6, *P*<0.001) (Fig. 5F). The above results implied that ROCK1 was a target of miR-335-5p and circ_0064288 elevated ROCK1 expression by targeting miR-335-5p in HCC cells.

## Discussion

CircRNA can affect gene expression through different mechanisms [13, 14]. In the nucleus, because circRNAs have repetitive sequences with parental DNA, when the accumulation of circRNAs in the nucleus increases, the circRNAs that remain in the nucleus will directly block the transcription process by forming RNA-DNA heterodimeric chains with parental DNA in a “negative feedback” manner, thus altering gene expression [15]. In the cytoplasm, circRNAs can modulate downstream genes’ expression by working as the molecular sponge for miRNAs [16]. Many circRNAs are identified as ceRNAs in cancer research. For instance, circ_0003998 may work as a ceRNA for miR-143-3p to attenuate the inhibitory effect of miR-143-3p on the epithelial-mesenchymal transition (EMT)-related regulator, FOS like 2, AP-1 transcription factor subunit [17]. In this work, circ_0064288 expression was revealed to be remarkably up-modulated in HCC. High circ_0064288 expression implied shorter overall survival time of HCC patients. Additionally, circ_0064288 could regulate the growth and metastatic potential of HCC cells. Considering circRNA has more stability than liner non-coding RNA, and it can be detected in not only tumor tissues, but also body fluids such as urine and blood, it is regarded as promising biomarkers for human diseases [18]. Our study suggest that circ_0064288 is a potential biomarker to evaluate the prognosis of HCC patients.

MiRNAs are key regulators of gene expression [19]. The aberrant expression of miRNAs is closely related to the progression of human malignancies. For example, miR-218 restrains the EMT process of HCC cells by targeting serpin mRNA binding protein 1 [20]. MiR-517a enhances the Warburg effect and growth of HCC cells by targeting fructose-1,6-bisphosphatase [21]. Reportedly, miR-335-5p is under-expressed and exerts tumor-suppressive effects in different human cancers. For instance, miR-335-5p impedes the growth, migration and invasion of colorectal cancer cells through down-modulation of lactic dehydrogenase B [22]. MiR-335-5p is also reported to remarkably restrain HCC progression in a previous study [23]. In this work, miR-335-5p was unveiled to be under-expressed in HCC tissues, miR-335-5p was validated to be a target of circ_0064288, and miR-335-5p counteracted the promoting effect of circ_0064288 on the malignancy of HCC cells.

Rho-associated protein kinase (ROCK), which is composed of ROCK1 and ROCK2, is implicated in modulating the movement of cells by functioning on the cytoskeleton [24, 25]. Reportedly, ROCK1 is also an essential player in regulating cell viability, motility and angiogenesis [26]. ROCK1 overexpression / activation is commonly linked to a more highly metastatic and aggressive phenotype of human cancers. ROCK1 overexpression is reported in many different types of cancer, including glioblastoma [27], melanoma [28], osteosarcoma [29], prostate cancer [30] and HCC [31]. Importantly, ROCK1 works as an oncogene in HCC, and it promotes HCC progression [31, 32]. In this work, ROCK1 was identified as a target of miR-335-5p, circ_0064288 augmented ROCK1 expression through competitive binding with miR-335-5p. Our data suggest that circ_0064288 / miR-335-5p axis is crucial to regulate ROCK1 expression in HCC, and for the first time, a ceRNA network consisting of circ_0064288, miR-335-5p, ROCK1 is presented.

There are some limitations in the present work. First of all, the bioinformatics analysis identified 72 up-regulated circRNAs in HCC, and only circ_0064288 was investigated in this study. The other 71 up-regulated circRNAs and the down-regulated circRNAs may also participate in HCC progression; factually, due to the different platform of microarray and the heterogeneity of HCC samples, different studies usually obtain different differentially expressed circRNAs, and there is a long way to fully clarify the role of circRNAs in HCC progression. Secondly, miR-335-5p has multiple potential downstream genes, not only ROCK1, implying that circ_0064288 / miR-335-5p axis can probably exert its biological function via other downstream mechanisms, which remain to be clarified in the following work. Last but not least, only in vitro experiments were designed in the present work, and in vivo models can further consolidate our conclusion.

## Conclusion

**Fig. 6:**
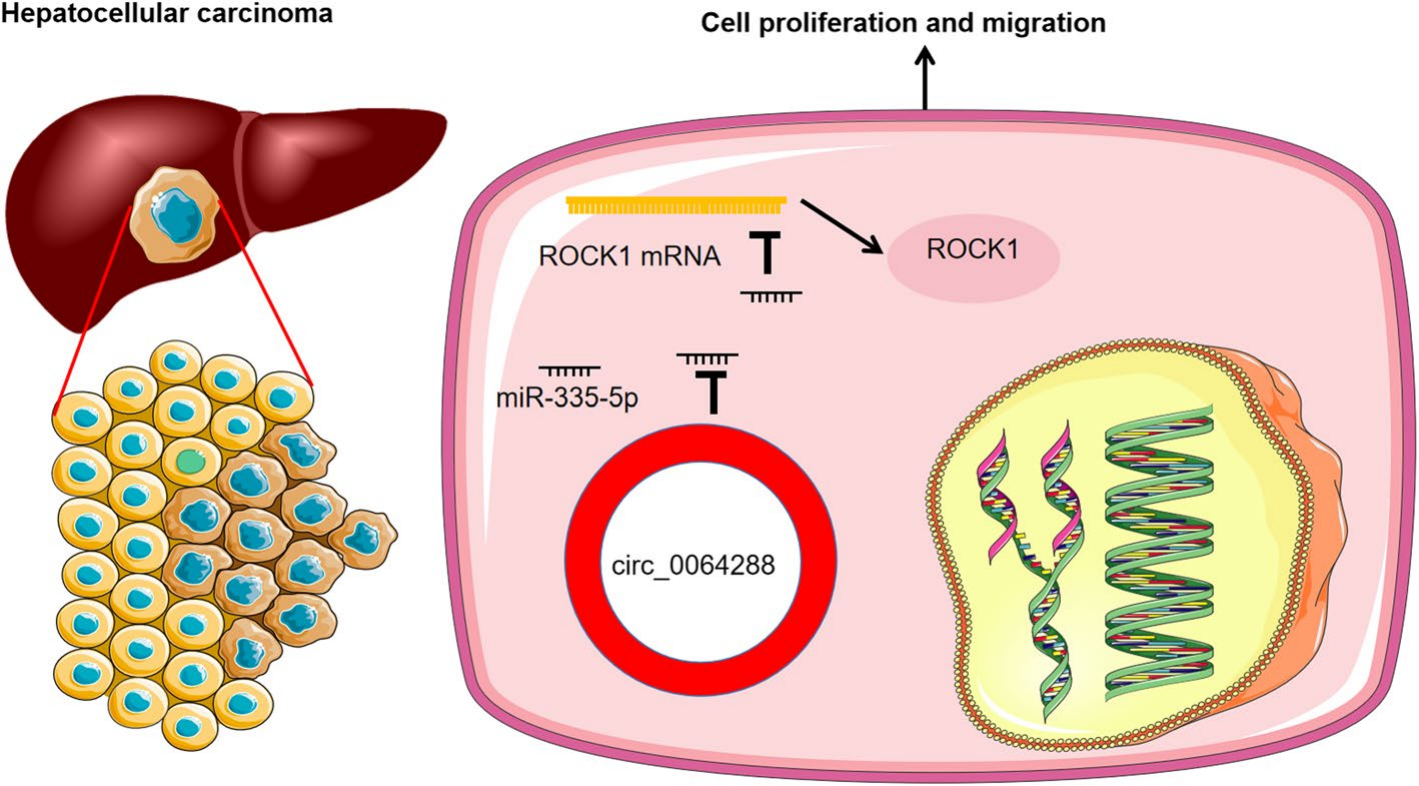
Graphic Abstract: In HCC cells, circ_0064299 represses the expression of miR-335-5p expression and up-regulates ROCK1 expression, to promote the malignancy of cancer cells, facilitating the proliferation and migration

In conclusion, this work reports that circ_0064288 expression is up-modulated in HCC and miR-335-5p expression was down-modulated in HCC. Circ_0064288 elevates ROCK1 expression by working as a sponge for miR-335-5p, thereby enhancing the malignant phenotype of HCC cells (Fig. 6), which provides a novel idea for the diagnosis and therapy of HCC.

## Supplementary Information

**Additional file 1.** Supplementary Table 1.

**Additional file 2.** Supplementary Table 2.

**Additional file 3.** Supplementary Table 3.

**Additional file 4.** Supplementary Figures.

## Data Availability

The datasets generated and/or analysed during the current study are available in the GEO datasets repository, [https://www.ncbi.nlm.nih.gov/geo/query/acc.cgi?acc=GSE94508, https://www.ncbi.nlm.nih.gov/geo/query/acc.cgi?acc=GSE97332].

## Abbreviations

ATCC: American Type Culture Collection
ATG7: Autophagy related 7
ceRNA: Competitive endogenous RNA
CCK-8: Cell counting kit-8
circRNA: Circular RNA
Circ_0064288: Circular RNA_0064288
EMT: Epithelial-mesenchymal transition
FBS: Fetal bovine serum
GEO: Gene Expression Omnibus
HCC: Hepatocellular carcinoma
miRNA: MicroRNA
miR-335-5p: MicroRNA-335-5p
MUT: Mutant type
PVDF: Polyvinylidene fluoride
qRT-PCR: Quantitative real-time polymerase chain reaction
ROCK1: Rho associated coiled-coil containing protein kinase 1
SDS-PAGE: Sodium dodecyl sulfate polyacrylamide gel electrophoresis
WT: Wild type
