# Supplementary material for: Circ_0064288 acts as an oncogene of hepatocellular carcinoma cells by inhibiting miR-335-5p expression and promoting ROCK1 expression
Source: BMC Cancer. 2022 Mar 14;22:265. doi: 10.1186/s12885-022-09323-8 (PMC8919637; doi:10.1186/s12885-022-09323-8)
Supplement: Supplementary file 4 — Additional file 4. Supplementary Figures. [file 12885_2022_9323_MOESM4_ESM.docx]

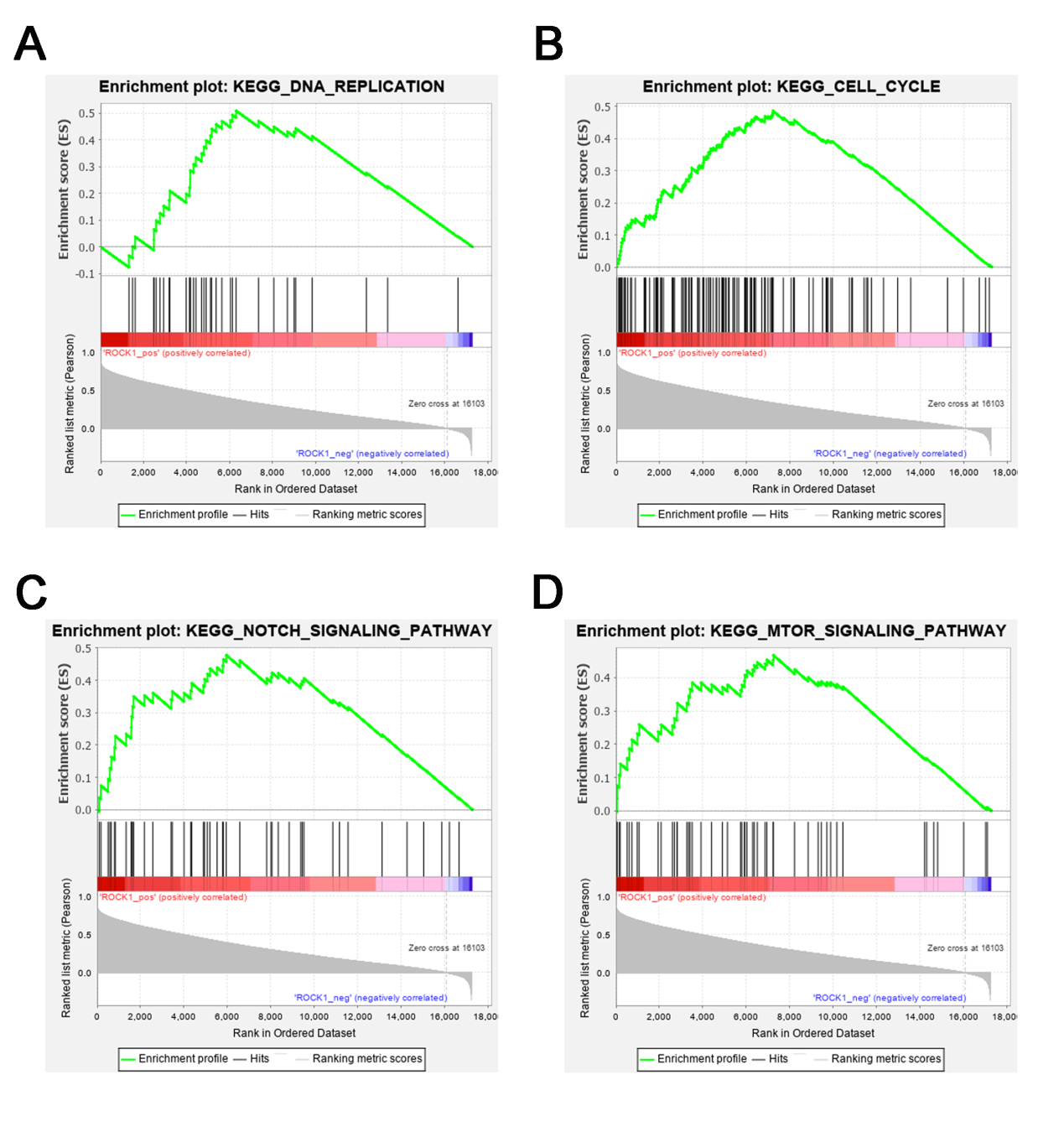


**Supplementary Figure 1. The relationship between the expression of ROCK1 and multiple signal pathways**

GSEA plot showing that ROCK1 overexpression is negatively associated with the multiple signal pathways / biological processes, including DNA replication (A), cell cycle progression (B), Notch signaling (C) and mTOR signaling (D). In all of the figures, *P* < 0.001, and FDR < 0.25.
